# Identification of Carboxylesterase Genes Implicated in Temephos Resistance in the Dengue Vector *Aedes aegypti*

**DOI:** 10.1371/journal.pntd.0002743

**Published:** 2014-03-20

**Authors:** Rodolphe Poupardin, Wannaporn Srisukontarat, Cristina Yunta, Hilary Ranson

**Affiliations:** Department of Vector Biology, Liverpool School of Tropical Medicine, Liverpool, United Kingdom; University of Perugia, Italy

## Abstract

**Background:**

Thailand is currently experiencing one of its worst dengue outbreaks in decades. As in most countries where this disease is endemic, dengue control in Thailand is largely reliant on the use of insecticides targeting both immature and adult stages of the *Aedes* mosquito, with the organophosphate insecticide, temephos, being the insecticide of choice for attacking the mosquito larvae. Resistance to temephos was first detected in *Aedes aegypti* larvae in Thailand approximately 25 years ago but the mechanism responsible for this resistance has not been determined.

**Principal Findings:**

Bioassays on *Ae. aegypti* larvae from Thailand detected temephos resistance ratios ranging from 3.5 fold in Chiang Mai to nearly 10 fold in Nakhon Sawan (NS) province. Synergist and biochemical assays suggested a role for increased carboxylesterase (CCE) activities in conferring temephos resistance in the NS population and microarray analysis revealed that the CCE gene, *CCEae3a*, was upregulated more than 60 fold in the NS population compared to the susceptible population. Upregulation of *CCEae3a* was shown to be partially due to gene duplication. Another CCE gene, *CCEae6a*, was also highly regulated in both comparisons. Sequencing and *in silico* structure prediction of CCEae3a showed that several amino acid polymorphisms in the NS population may also play a role in the increased resistance phenotype.

**Significance:**

Carboxylesterases have previously been implicated in conferring temephos resistance in *Ae aegypti* but the specific member(s) of this family responsible for this phenotype have not been identified. The identification of a strong candidate is an important step in the development of new molecular diagnostic tools for management of temephos resistant populations and thus improved control of dengue.

## Author Summary

Temephos is the most important insecticide used in larviciding campaigns to reduce the risk of dengue transmission. This organophosphate insecticide has been in use for over 50 years and resistance to this chemical has been reported in *Aedes aegypti* populations from Latin America, the Caribbean and from Asia. In other insect species, organophosphate resistance is typically associated with mutations in the target site, acetylcholinesterase, that decrease the insect's sensitivity to the insecticide, or increases in the activity of one or more carboxylesterase enzymes, either by overproduction and/or amino acid substitutions, that reduce the amount of insecticide reaching the target site. Neither of these mechanisms has been previously characterised at the molecular level in dengue vectors. Here we identify an *Ae aegypti* carboxylesterase gene with expression levels and amino acid sequence polymorphisms correlating with temephos resistance in Thailand. This is a key step in the development of tools to manage resistance in this mosquito species.

## Introduction

*Aedes aegypti* is a major vector of dengue fever and yellow fever viruses. Despite an effective vaccine, there are over 200,000 cases of yellow fever each year (WHO source, 2012). With no vaccine currently available for dengue, and no specific drug treatment, approximately 40% of the world's population is at risk of dengue fever and there may be as many as 390 million dengue infections per year [1].

Dengue is endemic in Thailand with the most severe manifestation of dengue, dengue haemorrhagic fever first reported in 1958 [2]. The number of dengue cases has been steadily increasing since 2009 with over 81,000 cases already reported in the first 7 months of 2013 and, predictions of between 100,000 and 120,000 cases for the whole year (Department of Disease Control, Thailand Ministry of Public Health, http://www.ddc.moph.go.th/).

Maintaining *Ae. aegypti* populations at low levels is crucial for dengue control in Thailand [3]. Environmental management including educational campaigns to remove unnecessary sources of standing water, coupled with covering of permanent water storage vesicles, is recommended to help reduce *Aedes* populations [4] but this is supplemented by the use of chemical insecticides. In Thailand, adult mosquitoes are predominately targeted with pyrethroid insecticides [5], mainly through the distribution of pyrethroid impregnated materials and the Ultra-Low-Volume (ULV) applications of pyrethroids [6]. Larval control primarily utilises the organophosphate insecticide, temephos, (Department of Disease Control, Thailand Ministry of Public Health) despite the known existence of temephos resistant populations of *Ae. aegypti* in many regions of Thailand [7], [8].

An understanding of insecticide resistance mechanisms is important for the development of tools and practices that can improve resistance management and thereby the sustainability of control interventions. In many insect species, organophosphate and carbamate resistance is caused by amino acid substitutions in the target site, acetylcholinesterase (ace-1), which reduces the sensitivity of this enzyme to the insecticide. The most common ace-1 substitution in mosquitoes occurs at amino acid residue 119 where the wild type glycine is substituted to serine [9]. However, in *Ae aegypti*, the codon usage at Glycine 119 makes this substitution very unlikely to occur [10]. Indeed, despite numerous reports of temephos resistance in *Ae aegypti* populations across the tropics, including at least one report of insensitive AchE [11], no target site mutations linked to organophosphate resistance have been detected to date. Organophosphate resistance can also be caused by elevated levels of esterase enzymes that can both act to sequester the insecticide, reducing the amount of active insecticide that reaches the target site [11], or to increase the rate of turnover of insecticide, by amino acid substitutions in the coding sequences of one or more esterases [12].

Elevated CCE activity has been associated with temephos resistance in several populations of *Ae aegypti* [13], [14], [15], [16], [17]. A small number of studies [16], [17], [18] have used microarray based approaches to detect genes associated with the resistance phenotype. Although several transcripts of detoxification genes were found to be evelated in temephos resistant populations (including *CCEae3a*, *CYP6Z8* and *CYP9M9*), a single clear candidate did not emerge from these studies. The current study provides evidence for elevated CCE activity in a temephos resistant population from Thailand and identifies a clear candidate gene that shows both elevated expression and amino acid polymorphisms in temephos resistant populations. Additional genes, potentially involved in temephos and/or permethrin resistance in *Ae aegypti* larvae are identified and discussed.

## Materials and Methods

### Study sites

Mosquito eggs were collected from four sites of Thailand including Chiang Mai (North, 18°47′25″N 98°59′4″E, 31^st^ October 2011), Nakhon Sawan (central, site 1 : 15°20′45″N 100°29′41″E, 5^th^ March 2012, site 2: 15°52′52″N 100°18′9″E, 27^th^ March 2012) and Phatthalung (south, 7°37′6″N 100°4′24″E, 2^th^ September 2011) (Figure S1). They were chosen based on previous reports of temephos resistance in these districts [8], [19], [20].

### *Aedes aegypti* collections

*Aedes aegypti* eggs from Phatthalung and Chiang Mai were collected using modified ovitraps by entomologists from the Department of Disease Control (Ministry of Public Health, Thailand). Eggs from Nakhon Sawan sites were collected by entomologists from office of Disease Prevention & Control 8 (DPC8, Nakhon Sawan). The modified ovitraps consisted of a dark plastic cup with a piece of filter paper over the inner part of the cup and filled with tap water. They were placed in the resting sites of *Ae aegypti* such as under sinks, beds, cupboards or any cool, humid and dark areas in and around the house. Eggs were then sent to the Liverpool School of Tropical Medicine (LSTM) where they were hatched in distilled water and reared in standard insectary conditions (temperature: 28+/−1°C; relative humidity: 75+/−5%; photoperiod: 12 hours day/night). An insecticide susceptible laboratory colony, New Orleans (NO) strain was used as control in the study. This population was originally collected in the namesake city located in Louisiana, United States.

### Insecticide susceptibility tests and synergist assays

Standard WHO larval bioassays were conducted to detect the level of susceptibility to temephos [21]. Bioassays were done on late 3rd/early 4th instar larvae using a range of seven temephos (Pestanal, analytic standard, diluted in ethanol) concentrations. Concentrations of insecticides were chosen in order to cover larval mortality range (0–100%). Three replicates of 20 larvae were used for each concentration and 1 ml ethanol was added in control cups. Mortality was recorded after 24 hours of exposure. Larval bioassays using permethrin were also performed to look for any evidence of cross resistance between insecticide classes.

Synergist bioassays were performed on the populations showing the highest temephos resistance levels using a cytochrome P450 inhibitor, piperonylbutoxide (PBO) at 0.3 ppm (piperonylbutoxide 90%, Sigma Aldrich, Inc., Italy), a glutathione S-transferase inhibitor, diethyl maleate (DEM) at 1 ppm (diethyl maleate >97.0% (GC), Sigma Aldrich Chemie GmbH, Austria) and a carboxylesterase inhibitor, S,S,S-tributylphosphorotrithioate (DEF) at 0.5 ppm (S.S.S-tributylphosphorotrithioate 98.1%, Chem service, Inc., USA). Inhibitors were mixed with insecticide dilutions in ethanol and 1 ml of the mixture was added to 99 ml of water according to the protocol of [22]. Different concentrations of synergists were previously tested in order to establish appropriate sub-lethal concentrations [22]. PBO was also used as a synergist in permethrin bioassays. To determine the LC50s and confidence intervals data were analyzed using a Probit model on R software [23].

### Measurement of carboxylesterase activities

Activity levels of α esterases and β esterases were measured in the Nakhon Sawan 2 population (NS2), which showed the highest resistance ratio to temephos, and in Phatthalung, the population most susceptible to temephos and permethrin. Procedures were based on mosquito-specific biochemical assay protocols [24], [25], [26]. Briefly, 15 larvae from NS2 and Phatthalung were individually homogenized in 3 mL of 0.01 M potassium phosphate buffer (KPO_4_), ph 7.2 and 100 µl of each sample homogenate were then transferred by triplicate to a 96-well microtiter plate. Then, 100 µl of α/β naphthyl acetate (3 mM) were added to each well, followed by 15 minute incubation at room temperature. Finally, 100 µl of dianizidine (4 mM) were added, followed by 4 minute incubation, and then absorbance was read at a wavelength of 540 nm. Absorbance values where normalized by measuring protein content using a Bradford assay according to manufacturer's protocol (Sigma, St Louis, MO). Data significance was compared using a Mann-Whitney test (N = 15).

### RNA extractions and labeled cRNA synthesis

The most resistant population Nakhon Sawan 2 was chosen for the microarray experiment. Phatthalung was used as susceptible population because of its geographical proximity (Figure S1). Three groups of early 4th instar larvae (15 larvae each) were used for total RNA extractions: Phatthalung (P), Nakhon Sawan 2 unexposed (NS 2 Unexp) and Nakhon Sawan 2 larvae (NS 2 Exp) which survived a temephos bioassay inducing 60% mortality (24 hour exposure to 0.032 ppm temephos). Surviving larvae were left to recover in clean water for 24 hours after exposure to reduce the impact of short term gene induction on the transcriptomic profile. The Arcturus Picopure RNA Extraction Kit (Arcturus, California, USA) was used according to the manufacturer's protocol and 100 ng total RNA per biological replicate were amplified and labelled with Cy-5 and Cy-3 dyes with the ‘Two colors low input Quick Amp labeling kit’ (Agilent technologies, Santa Clara, CA, USA) according to manufacturer's instructions. Labelled cRNA were purified with the Qiagen RNeasy kit (Qiagen, Hilden, Germany). Quantification and quality assessment of labeled cRNA were performed with the Nanodrop ND-1000 (Thermo Scientific, DE, USA) and the Agilent 2100 Bioanalyser (Agilent Technologies).

### Hybridizations, data acquisition and statistical analysis

Microarray hybridizations were performed with the 15 k Agilent “*Aedes* microarray” (ArrayExpress accession number A-MEXP-1966), containing eight replicated arrays of 60-mers oligo-probes representing 14,204 different *Ae. aegypti* transcripts from AaegL1.2 Vectorbase annotation and several control probes. For each comparison, five hybridizations were performed including two dye-swaps in which the Cy3 and Cy5 labels were swapped between samples. After 17 h hybridization, non-specific probes were washed off with the Agilent microarray hybridization kit according to manufacturer's instructions. Slides were scanned immediately with an Agilent G2205B microarray scanner. Spot finding and signal quantification for both dye channels were performed using the Agilent Feature Extraction software (Agilent Technologies). Data were then loaded into Genespring GX (Agilent Technologies) for normalization and statistical analyses. For each population comparison, only transcripts flagged ‘present or marginal’ in four of five hybridizations were used for further statistical analysis. Mean transcription ratios were then submitted to a one sample Student's t-test (N = 3) against the baseline value of 1 (equal transcription level in both populations) with Benjamini and Hochberg's multiple testing correction. For each selected population, transcripts showing a >2 fold change in either direction and a t-test P-value lower than P<0.01 after multiple testing correction were considered significantly differentially transcribed compared to the susceptible population. Descriptions and GO-terms of transcript-IDs were extracted from VectorBase (www.vectorbase.org) using BIOMART and completed with Blast2GO software (BioBam Bioinformatics S.L. (Valencia, Spain)). GO term Enrichment analysis was performed on the significant up-regulated genes found in both comparisons “NS2 exp vs P” and “NS2 Unexp vs P” using Blast2GO software and Fisher's exact test with FDR<0.05 according to [27]. All microarray data were uploaded to Arrayexpress (E-MTAB-1934, www.ebi.ac.uk/arrayexpress/).

### Microarray data validation by RT-qPCR

Transcription levels of six genes (four P450s, one CCE and one ABC transporter) found significantly differentially transcribed in at least two comparisons were validated by reverse transcription followed by real-time quantitative PCR (RT-qPCR) as described in [28]. As a secondary control, the susceptible New-Orleans (NO) population was included. Two micrograms of total RNA per biological replicate were treated with DNAse I (Invitrogen, Carlsbad, CA, USA) and used for cDNA synthesis with superscript III and Oligo-dT20 primer (Invitrogen) according to manufacturer's instructions and resulting cDNAs were diluted 50 fold. Real time quantitative PCR reactions of 25 µL were performed on a MX3005P qPCR machine (Agilent technologies, CA, USA) using Brilliant III ultrafast SYBR green mastermix (Agilent technologies, CA, USA), 0.3 mM of each primer and 5 µL of diluted cDNAs. A melt curve analysis was performed to check for the unique presence of the targeted PCR product. Quantification of transcription level was performed according to the ΔΔCt method taking into account PCR efficiency [29] and using two housekeeping genes for normalization: the ribosomal proteins L8 (AAEL000987) and S7 (AAEL009496). Results were expressed as mean transcription ratio (±95% confidence intervals) between Nakhon Sawan 2 and the susceptible populations New Orleans and Phatthalung. All primer sequences are included in supplementary table S5.

### *CCEae3a* gene copy number analysis

Three different groups of 4^th^ instar larvae were used: P, NS2 unexposed and NS2 exposed mosquitoes. NS2 exposed mosquitoes were survivors of a temephos bioassay inducing more than 80% mortality after 24 hours. Genomic DNAs were extracted from 8 individual larvae per group using DNeasy Blood and Tissue Kit according to manufacturer's instructions (Qiagen, Hilden, Germany) and were treated with RNAse A (Qiagen, Hilden, Germany) to remove any RNA contaminants. DNA quantities were assessed on a Nanodrop ND-1000 spectrophotometer. Quantitative PCR reactions were performed as described above on *CCEae3a* gene (same primers used above) with *AAEL000987* (RPL8) and *AAEL012167* (Elongation factor) (see table S5 for primer sequences) as housekeeping genes. The relative copy number fold-change was calculated using the 2−ΔΔCt method.

### *CCEae3a* cDNA sequencing

To identify any amino acid polymorphisms that might be associated with temephos resistance, sequencing of *CCEae3a* cDNA sequence was performed on Nakhon Sawan 2 larvae which survived a concentration of temephos inducing 90% mortality and on unexposed Phatthalung larvae. Total RNAs from 10 individual larvae were extracted using Trizol according to the manufacturer's instructions (Invitrogen, Carlsbad, USA) and total RNA quantities were assessed using a Nanodrop ND-1000 (Thermo Scientific). Genomic DNA contaminants were then digested using DNase I (Invitrogen) and total RNAs were reverse transcribed according to the same protocol used for qPCR validation. Primers were designed (Table S1) to amplify the whole *CCEae3a* sequence available on Vectorbase (AAEL005112-RA, www.vectorbase.org). PCR amplification was carried using Phusion High-Fidelity DNA Polymerase (Thermo Scientific) using the following conditions: Initial denaturation at 98°C for 30 seconds followed by 35 cycles of 10 sec denaturing at 98°C, 20 sec annealing at 66°C and one minute extension at 72°C. Last extension step 72°C last during 10 min. PCR products were visualized on a 1% agarose gel and purified using a GeneJET Gel Extraction Kit (Fermentas, Vilnius, Lithuania). The PCR products were cloned into DH5 competent cells using pJET 1.2/blunt Cloning Vector kit (Fermentas, Vilnius, Lithuania). Plasmids were extracted using GeneJET Plasmid Miniprep Kit, (Fermentas) and sequenced (Macrogen, Amsterdam, the Netherlands) using pJET primers and two internal primers (Table S5).

### *In silico* structure prediction of CCEae3a

The secondary structure and three-dimensional structure of the different polymorphic variants of CCEae3a were predicted by the Protein Homology/analogY Recognition Engine (PHYRE2) (Structural Bioinformatics Group, Imperial College, London). This method uses structural alignments of homologous proteins of similar three-dimensional structure in the structural classification of protein databases to obtain a structural equivalence of residues. The top 20 highest scoring matches of the query to known template structures are used to construct 3D model of the query.

## Results

### Insecticides susceptibility tests and synergist assays

The Phatthalung (P) populations showed the lowest LC_50_ to temephos and resistance ratios were calculated compared to this population, and according to the standard laboratory susceptible New Orleans (NO). NS 2 showed the highest resistance to temephos (RR at LC50 = 5.9 –9.85 fold) followed by NS 1 (RR at LC50 = 3.3–5.5 fold) and CM (RR at LC50 = 2.1–3.5 fold) (Table 1). Larval bioassays using permethrin showed much higher LC50s in both NS1 and NS2 populations compared to P (RR at LC50 = 29.1 and 31 fold respectively) and intermediate LC50 in the CM population (RR = 8.2 fold) (Table 1). Although permethrin larval bioassays were not performed on a standard lab susceptible strain in this study, two previous studies have reported lab susceptible LC50 for permethrin as approximately 0.0007 ppm [8], [30] which is similar to the 0.0005 value obtained for the P population in the current study.

**Table 1:** Susceptibility of *Ae. aegypti* larvae from Thailand to temephos and permethrin with or without synergists.

			Confidence intervals (95%)				
Strains	Treatments	LC_50_ (ppm)	Lower	Upper	RR_(P)_	RR_(NO)_	SR
New Orleans (NO)	temephos	0.004	0.0036	0.0045	1.67	1.00	-
Phatthalung (P)	temephos	0.0024	0.0022	0.0026	1	0.60	-
Chiang Mai (CM)	temephos	0.0084	0.0076	0.0093	3.5	2.10	-
Nakhon Sawan 1 (NS 1)	temephos	0.0132	0.0121	0.0142	5.5	3.30	-
	temephos+PBO	0.0175	0.0153	0.0199	7.31	4.38	0.75 *
	temephos+DEM	0.0119	0.0099	0.0143	4.97	2.98	1.11
	temephos+DEF	0.0042	0.0033	0.0052	1.75	1.05	3.14 *
Nakhon Sawan 2 (NS 2)	temephos	0.0236	0.021	0.0264	9.85	5.90	-
	temephos+PBO	0.0207	0.0189	0.0227	8.65	5.18	1.14
	temephos+DEM	0.0196	0.0181	0.0213	8.19	4.90	1.20
	temephos+DEF	0.0095	0.0084	0.0107	3.96	2.38	2.48 *
Phatthalung (P)	permethrin	0.0005	0.0004	0.0005	1	-	-
Chiang Mai (CM)	permethrin	0.0039	0.0034	0.0045	8.22	-	-
Nakhon Sawan 1 (NS 1)	permethrin	0.0148	0.013	0.0168	30.97	-	-
Nakhon Sawan 2 (NS 2)	permethrin	0.0139	0.0127	0.0152	29.14	-	-
	permethrin + PBO	0.006	0.0054	0.0067	12.6	-	2.32 *

LC_50_: Lethal concentration at which 50% of the population is killed; Resistance ratios RR_(P)_ are all compared to Phatthalung while RR_(NO)_ are compared to NO. Synergist ratios are the ratio between the LC_50_ of the treatment without synergist and the LC_50_ using each synergist and significant values are marked with *. The synergists used were the P450 inhibitor PBO (piperonyl butoxide, 5-((2-(2-butoxyethoxy) ethoxy) methyl)-6-propyl-1,3-benzodiox-ole), the GST inhibitor DEM (diethyl maleate), and the CCE inhibitor DEF (S,S,S-tributyl phosphorotrithioate).

Synergist bioassays were performed on both NS 1 and NS 2 populations. The use of temephos + PBO or DEM had no significant effect on NS 1 and NS 2 compared to temephos treatment alone. However, the DEF treatment significantly improved the toxicity of temephos by 3.14 fold in NS 1 and 2.48 fold in NS 2 compared to temephos alone. Finally the use of PBO+permethrin in combination showed an improved efficacy by more than two fold in NS 2 larvae compared to permethrin alone.

### Measurement of carboxylesterase activities

**Figure 1:**
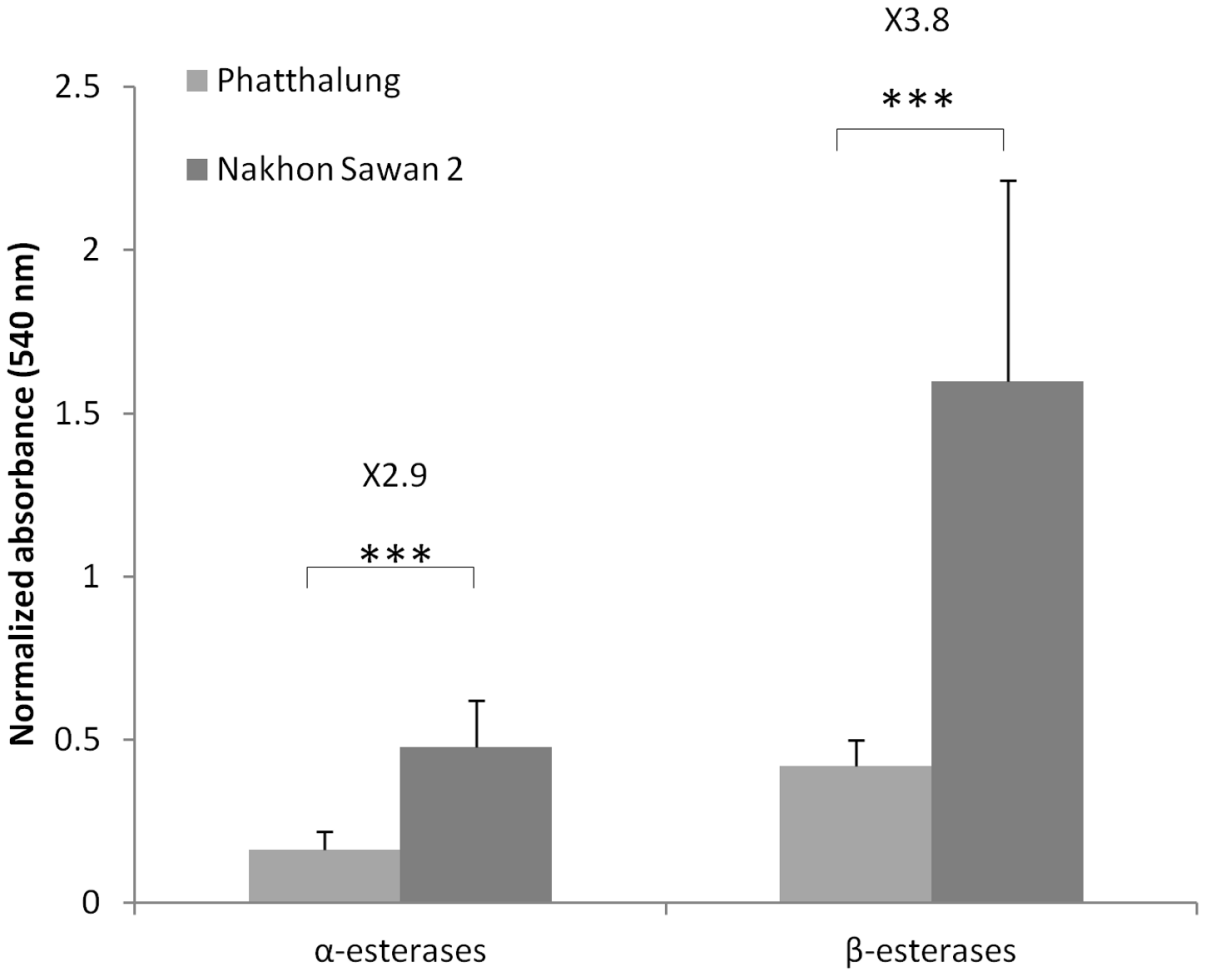
Comparison of esterase activities between Phatthalung and Nakhon Sawan 2 populations. Absorbance values were measured after 4(*** P-val<0.05, Mann-Whitney).

Comparison of constitutive detoxification enzyme activities between the susceptible population Phatthalung and the most insecticide-resistant population NS 2 revealed increased α- and β-carboxylesterase activities in NS 2 compared to P (2.9 fold and 3.8 fold with P<0.05) (Figure 1).

### Microarray analysis

**Figure 2:**
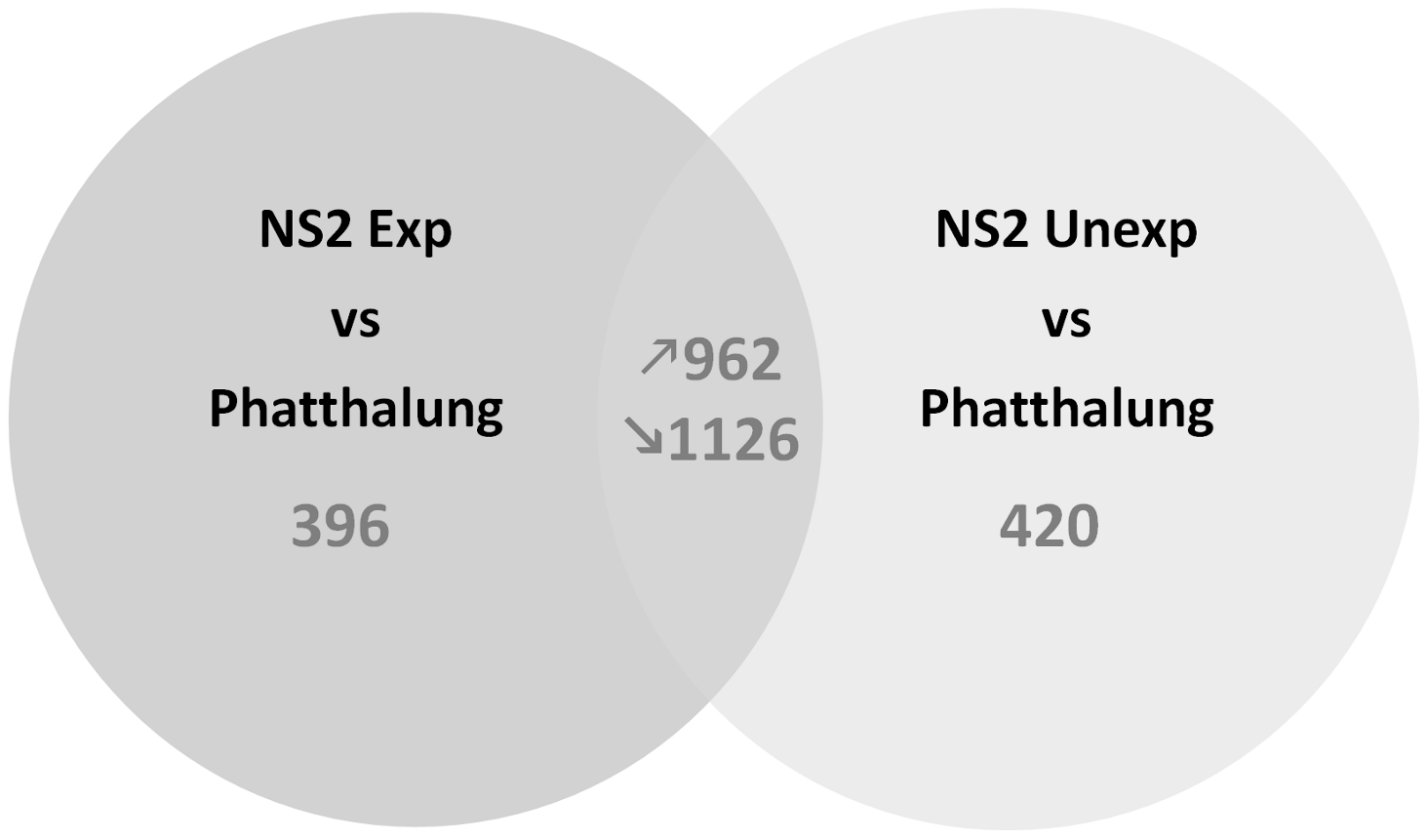
Summary of the genes differentially transcribed in the comparisons Nakhon Sawan 2 Unexp and Exp vs Phatthalung. The Venn diagram shows the number of genes found significantly (P value<0.01) over- or under-transcribed (>2 fold in either direction) in one or both comparisons. Upward arrows indicate over- transcribed in Nakhon Sawan 2 compared to Phatthalung, downward represent under-transcribed.

**Figure 3:**
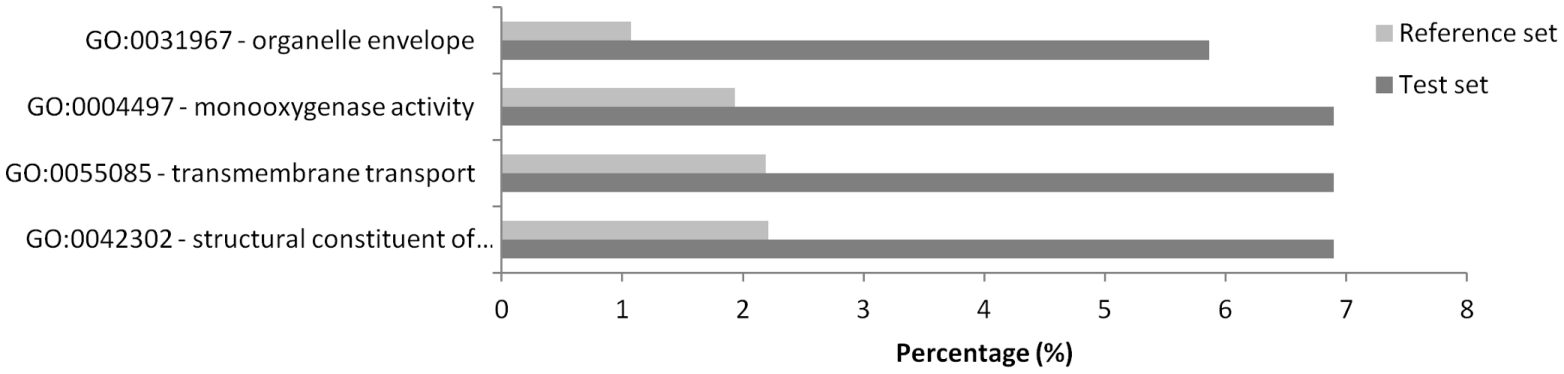
GO term enrichment analysis conducted on transcripts found upregulated in both comparisons “NS 2 Exp vs P” and NS 2 Unexp vs P”. GO-term categories represented were found significantly enriched compared to the reference set (all transcripts present on the microarray) after a Fisher's exact (Pval<0.01) with multiple testing correction. Test set percentage indicates the percentage of up regulated genes belonging to a GO term category compared to all up-regulated genes used in the GO-term analysis while the reference set percentage indicates the percentage of a particular GO-term category compared to all genes with GO-terms on the microarray.

By using a microarray approach, we detected 2484 transcripts significantly differentially regulated between NS2 Exp and Phatthalung, 2508 between NS2 Unexp and P and 0 between NS2 Exp and NS2 Unexp (RC) (Absolute change >2 fold, corrected P-value<0.01). Validation of microarray data on six selected genes by RT-qPCR revealed an acceptable correlation between transcription patterns obtained by the two techniques (mean R2 = 0.92) except for *CYP6Z9* for which transcription pattern among comparisons was not confirmed (Table S1). Between the comparisons “NS2 Exp vs P” and “NS2 Unexp vs P”, 2088 transcripts were commonly found differentially regulated, including 962 up- and 1126 down-regulated transcripts (Figure 2). Among these up-regulated transcripts, GO term Enrichment analysis revealed 8 GO terms over represented compared to the whole microarray (FDR<0.05), all linked with P450 activities (Figure 3a). Within the 962 up regulated transcripts found in both comparisons (Table S2), 42 *CYPs* were detected, 18 of which belong to the *CYP9J* family (Table S3).

**Figure 4:**
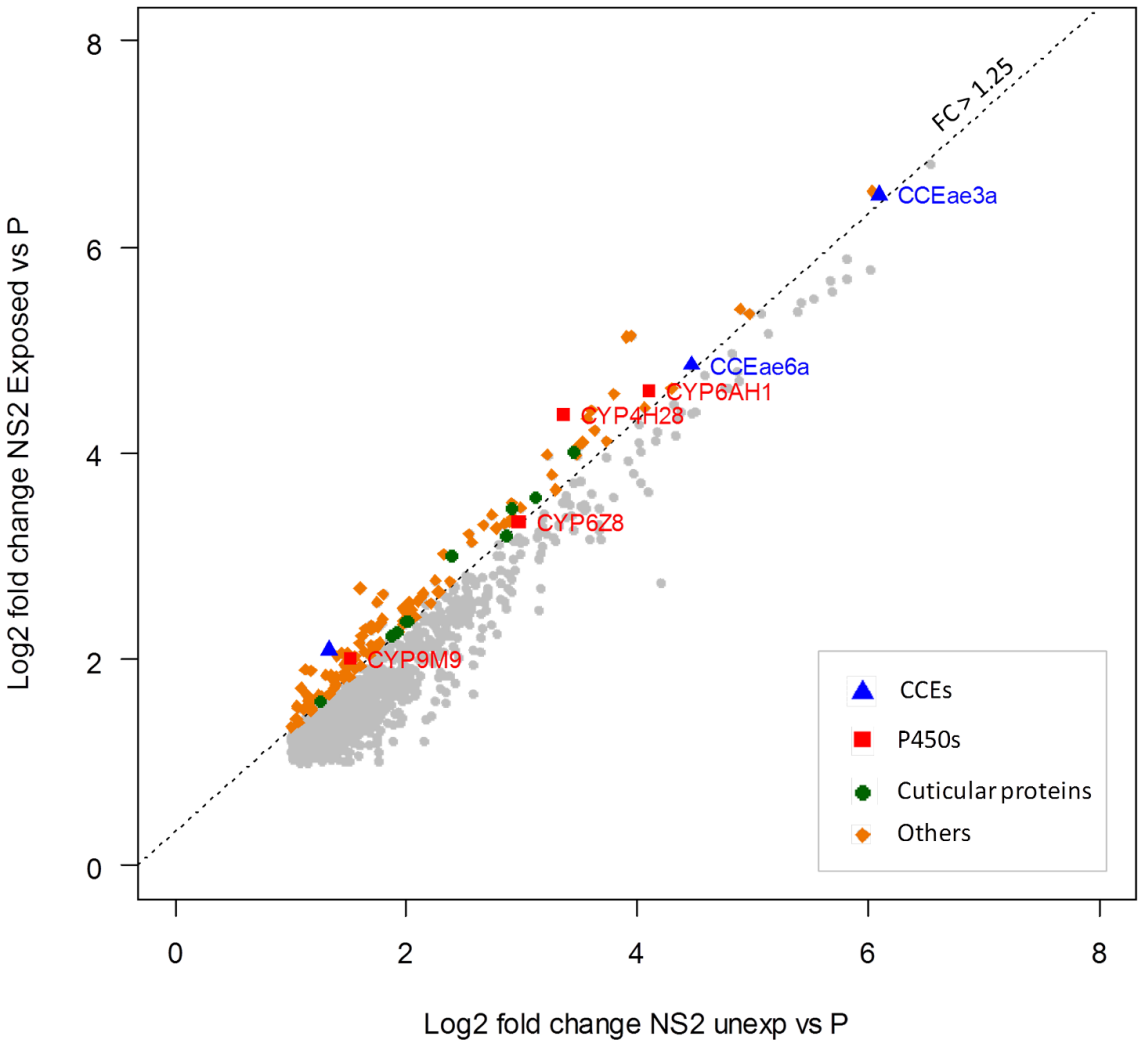
Significant up-regulated probes commonly found in NS2 Exp vs P and NS2 Unexp vs P. Colored probes correspond to the transcripts with fold changes 1.25 fold higher in NS 2 (exposed) vs Phatthalung compared to NS 2 (Unexposed) vs Phatthalung comparison.

Larvae from NS2 are resistant to both temephos and permethrin. In an attempt to prioritise genes putatively involved in temephos resistance we applied an additional layer of filtering to derive our candidate gene list. We specifically looked for genes whose fold change compared to the susceptible P population were higher in the NS2 surviving temephos exposure than in the unexposed NS2 vs P comparison. By using an arbitrary ratio threshold of 1.25, the candidate list was reduced to 122 transcripts (Table S4). This threshold was chosen in order to be within the range of differential detection of the microarray technology (in line with recommendations from Agilent Techonologies). These candidates are highlighted in the volcano plot (Figure 4) which also shows all transcripts significantly upregulated in Nakhon Sawan Unexp compared to Phatthalung. Interestingly, among the most overtranscribed genes figured one carboxylesterase *CCEae3a (AAEL005112)* which was overtranscribed around 60 fold in Nakhon Sawan Unexp compared to Phatthalung and 91 fold in Nakhon Sawan Exp compared to Phatthalung (ratio RS/RC = 1.33). Two other esterases were also found more upregulated in RS comparison compared to CS: CCEae6A (*AAEL015264-RA*) (29 fold upregulated in RS, 22 fold in CS) and CCEglt1K AAEL006097-RA (4.2 fold in RS, 2.5 fold in CS). Four cytochrome P450s were also present in the candidate genes list: *CYP6Z8* (*AAEL009131-RA*), *CYP9M9* (*AAEL001807-RA*), *CYP6AH1* (*AAEL007473-RA*) and *CYP4H28* (*AAEL003380-RA*). Multiple transcripts coding for cuticular proteins were also found significantly overtranscribed among the 122 transcripts, including 6 paralogous genes belonging to the CPLC group.

### *CCEae3a* gene copy number analysis

Quantitative PCR showed a significantly higher *CCEae3a* gene copy number in NS 2 unexposed (>165 fold, Pval<0.01) and NS2 Exposed (>350 fold, Pval<0.01) compared to Phatthalung strain (Figure S2).

### *CCEae3a* cDNA sequencing

**Figure 5:**
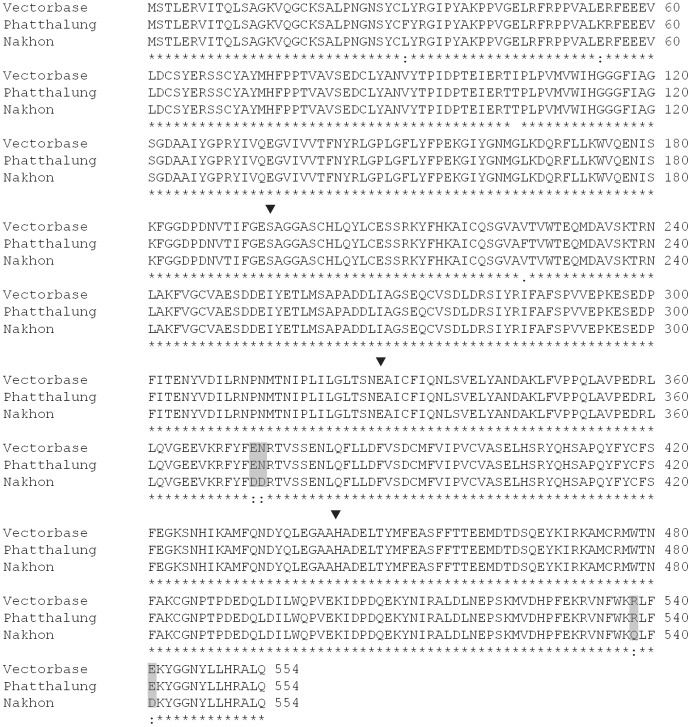
Partial alignment between three translated CCEae3a sequences: Two sequences from the susceptible strain Phatthalung and Vectorbase and one from the resistant strain Nakhon Sawan 2. Grey highlight shows the amino acid changes between the resistant and susceptible populations. Catalytic triad is highlighted by the symbol**▾**.

Sequencing of the cDNA sequence of *CCEae3a* (*AAEL005112-RA*) revealed the presence of non synonymous mutations between the sequences from Vectorbase, Phatthalung and the resistant population NS2. The derived amino acid sequence of NS2 had amino acid substitutions AAT positions 373 (GAA to GAC, leading to the change of an aspartic acid to glutamic acid), 374 (AAT to GAT, asparagine to glutamic acid), 538 (CGA to CAA, arginine to glutamine) and 541 (GAA to GAC, glutamic acid to aspartic acid) compared to Vectorbase and Phatthalung sequences (Figure 5).

### *In silico* structure prediction of CCEae3a

**Figure 6:**
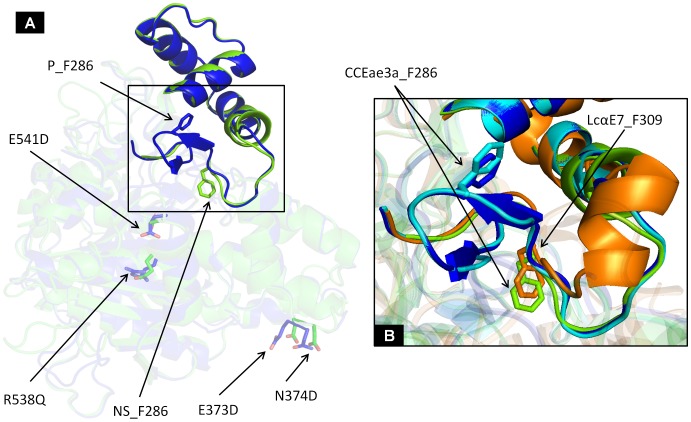
Analysis of CCEae3a 3D models. (A) CCEae3a_Nakhon Sawan (green) and CCEae3a_Phatthalung (blue) model superimposition shows no significant differences in the protein overall fold between resistant and susceptible alleles due to the polymorphic residues despite variations on the substrate binding site. (B) Close-up view of conformational differences at the substrate binding site between CCEae3a_Nakhon Sawan (green) and CCEae3a_Phatthalung (blue) revealed that F286, involved in the substrate recognition by homology with LcαE7 (orange), could be the key to explain both CCEae3a_Phatthalung and CCEae3a_vectorbase (cyan) susceptibility.

The models for Nakhon Sawan, Phatthalung, Vectorbase and mutated Vectorbase (Vectorbase sequence with the NS mutations at the positions 373, 374, 538 and 541) sequences were generated using PHYRE2 web server in the intensive mode. For all of them, 99% of the residues were modelled at more than 90% confidence in the final model and the best ranked match was the carboxylesterase αE7 from the Australian sheep blowfly *Lucilia cuprina* (LcαE7) with 34% identity. The *in silico* models enabled the polymorphic residues of the analysed variants (E373D, N374D, R538Q and E541D) to be localised and to identify those residues involved in the active site by homology with LcαE7. The most interesting difference between resistant and susceptible forms was found more than 20 Å away from the polymorphic residues and involved residues that belong to the putative substrate-binding site (Y283-G293) (Figure 6).

## Discussion

Previous studies have reported temephos resistant populations of *Ae aegypti* from Thailand [8], [19]. The objective of the current work was to identify the mechanism(s) responsible for this resistance. Bioassays were conducted on four populations of Thai mosquitoes and a susceptible laboratory population. Larvae from the P population from the southern Phatthalung province were fully susceptible to temephos with a lower LC50 than the New Orleans laboratory strain. Full susceptibility to temephos was also reported in the neighbouring province of Songkhla in 2005 [19]. Two other populations, NS1 and CM, showed low levels of temephos resistance (according to classifications in [31]) and one population, NS2, from central Thailand, showed medium levels of resistance, with RR from 6–10 fold. An earlier study also found the highest levels of temephos resistance in the Nakhon Sawan province [19] and the RRs obtained in the current study are similar to those reported from this province in a 2005 study, despite the use of different lab susceptible populations [8]. Although the current study did not directly assess the impact of the observed resistance on the field efficacy of temephos, earlier studies in Brazil clearly demonstrated an impact of resistance levels of similar magnitudes to the NS2 population on the duration of temephos efficacy in simulated field assays [32]. Hence it is likely that temephos resistance is compromising dengue control in central Thailand but, as noted by others [19], insecticide resistance in *Ae aegypti* appears to be very focal (note the marked differences in the Temephos LC50 between NS1 and NS2, separated by a distance of 60 Kms).

Permethrin resistance was also detected in *Ae aegypti* larvae from Chiang Mai and from both populations from Nakhon Sawan province. Again this agrees with earlier bioassays data from Thailand [8]. Pyrethroids are not directly applied as larvicides in Thailand but contamination of breeding sites may occur by the use of pyrethroids as aerial sprays to control dengue epidemics. Alternatively, the co-occurrence of both temephos and permethrin resistance in the same population may be caused by cross-resistance as was proposed following a temephos selection experiment in Cuba [33]. Possible mechanisms for this putative cross resistance are discussed below.

The data from enzyme inhibitors suggests that temephos resistance in the Nakhon Sawan province is linked to carboxylesterase activities. Conversely, the cytochrome P450 inhibitor, PBO, had the biggest impact on permethrin resistance in the NS2 population. However, even after addition of PBO, NS2 remained moderately resistant to permethrin suggesting that pyrethroid target site resistance may be present in the population: two sodium channel mutations associated with permethrin resistance, V1016G and F1534C are known to be widespread in Thailand [34], [35], [36], [37].

Further support for a key role for carboxylesterases in conferring temephos resistance is provided by biochemical assays using alpha- and beta-naphtylacetate as substrates. Significantly higher levels of esterase activity were detected in the NS2 population compared to the susceptible population from Southern Thailand (P). Again, this mimics findings from other temephos resistant populations [14], [15]. Although both changes in gene expression and allelic variation in individual CCE proteins has been associated with organophosphate resistance [38], [39] the latter is typically associated with a *decrease* in esterase activity, as measured with general esterase substrates [40], [41], [42]. We therefore hypothesised that one or more up-regulated carboxylesterase genes were responsible for the temephos resistance and thus used a microarray platform to identify transcripts that were upregulated in the resistant NS2 population compared to the susceptible Phatthalung population.

Phatthalung was used as a susceptible population, as opposed to a standard laboratory susceptible population, in an attempt to reduce the impact of extended laboratory colonisation and geographical differences on the transciptome data. It was therefore surprising to find over 2000 transcripts significantly differentially transcribed between the two Thai populations. In a three way comparison we compared both NS2 unexposed to insecticides and a subset of NS2 population that had survived temephos exposure and been sacrificed 24 hours after insecticide exposure with the Thai susceptible population. We did not observe any significant differences between the NS2 exposed and unexposed populations but we used these three data sets to filter our candidate list in two steps. Firstly we discarded genes that were only upregulated in the NS2 population in one of the comparisons (Figure 2) focusing initially on the subset of 962 transcripts that were commonly upregulated in the NS2 exposed vs P and the NS2 unexposed vs P. Interestingly, this subset of transcripts contained a large number of cytochrome P450 genes. This was confirmed by the enrichment analysis which showed a clear enrichment of GO terms linked with P450 activities in the overtranscribed genes compared to the whole microarray. Over half of the upregulated P450s belonged to the CYP9J family (Table S3). CYP9Js have been widely implicated in pyrethroid resistance in *Ae aegypti* populations across the globe [27], [43], [44], and several of these have been biochemically characterized and been shown to metabolize pyrethroids [45]. Further confirmation of the role of this P450 family in pyrethroid resistance comes from transgenic expression of CYP9J28 in *Drosophila melanogaster* which conferred an elevated level of resistance to pyrethroids [46].

To further refine our list of candidate genes responsible for temephos resistance, we hypothesised that genes putatively conferring this phenotype would exhibit a higher fold change differential in transcript levels in the NS2 exposed versus susceptible comparison than the NS2 unexposed vs susceptible. We therefore reduced our candidate list from 962 to 122 transcripts by dividing the fold changes in “NS2 Exp vs P” comparison by fold changes in “NS2 Unexp vs P” comparison and using an arbitrary cut off of >1.25. Only four cytochrome P450s remained in this refined candidate list (*CYP6Z8*, *CYP9M9*, *CYP6AH1*, *CYP4H28*), none of which belonged to the CYP9J family, perhaps indicating that the over expression of the CYP9J genes in NS2 contributes to the permethrin resistance phenotype but has a negligible role in conferring temephos resistance. CYP6Z8 has recently been shown to metabolize the 3-phenoxybenzoic alcohol (PBAlc) and 3-phenoxybenzaldehyde (PBAld), common metabolites produced by carboxylesterases [47], and it is possible that elevated levels of this enzyme is an important secondary resistance mechanism.

Three carboxylesterase genes were present within final candidate list. One of these (*AAEL006097-RA*) encodes a putative glutactin which, although potentially catalytically active as it contains the catalytic triad and oxyanion hole, is not thought to be involved in xenobiotic detoxification. The two remaining carboxylesterases *(CCEae3a (AAEL005112)* and *CCEae6A (AAEL015264))* belong to the alpha esterase clade, a group typically associated with dietary or xenobiotic detoxification functions. *CCEae3a* was overtranscribed more than 90 fold in NS2 exposed compared to Phatthalung and more than 60 fold in NS2 unexposed compared to P. To verify that this did not simply reflect an exceptionally low level expression in the southern Thai population, we also included the lab susceptible New Orleans in the qPCR. There was no significant difference in the expression of CCEae3A in the two susceptible populations (Table S1). CCEae6A was also highly over expressed in NS2 compared to the P population (29 fold in exposed, 22 fold in unexposed). Of these two alpha esterases, *CCEae3a* appears a particularly strong candidate for temephos resistance, as this gene is known to be overexpressed in temephos resistant populations from Martinique [16], [48] and Brazil [17]. Interestingly, the copy number of CCEae3a was much higher in the NS2 resistant strain than the susceptible P strain, and also elevated in the subset of the NS2 strain surviving temephos exposure compared to the general NS2 population. This suggests that the overtranscription of *CCEae3a* may at least be partly due to gene amplification, similar to the mechanism observed in *Culex pipiens* [11].

In Martinique Island, both *CYP6Z8* and *CCEae3a* were found upregulated together in pyrethroid and organophosphate resistant populations of *Aedes aegypti* [16], [48] supporting the possible coordinated role of CYP6Z8 and CCEae3a in insecticide detoxification [47].

In addition to the over expression of CCEae3a cDNA sequence, several non-synonymous mutations were found between the sequences from Phatthalung compared to Nakon Sawan 2. *In silico* structure predictions of *CCEae3a*, based on the carboxylesterase αE7 from the Australian sheep blowfly *Lucilia cuprina* (LcαE7) [49] predicted that the polymorphic residues were not adjacent to the insecticide binding site. Nevertheless, the resistant variants lacked the hairpin loop between Y283 and G293 which was found in the susceptible population. It is possible that this loop displaces the F286 residue (homolog to F309 in LcαE7) that seems to be essential in stabilizing OPs in the LcαE7 active site. Further work is needed however to determine whether the allelic variants differ in their enzymatic activity and if either or both forms are capable of sequestering and/or metabolising temephos.

Temephos is one of the key insecticides for dengue control across the tropics but operationally significant levels of resistance are being increasingly reported [18]. Carboxylesterases have long been suspected to play a key role in mediating this resistance but to date no clear candidates had been identified. The identification of strong candidate genes has now laid the foundations for the development of molecular diagnostics to assess the correlation between the overexpression of these genes and temephos resistance across the distribution of *Ae aegypti*.

## Supporting Information

Figure S1 **Sampling sites of** ***Aedes aegypti*** **mosquitoes in Thailand.** Eggs were collected from four different sites were used: Chiang Mai (CM) (Oct 2011), Nakhon Sawan 1 (NS 1) (March 2012), Nakhon Sawan 2 (NS 2) (March 2012), Phatthalung (P) (September 2012).
(TIF)

Figure S2 **CCEae3a gene copy number analysis.** qPCR was conducted on three batches of 8 individual 4th instar larvae gDNA from NS 2 unexposed, NS2 Exposed (larvae survivors of a temephos bioassay inducing more than 80% mortality after 24 hours) and Phatthalung (P). 95% confidence intervals were calculated for qPCR fold changes and a Mann-Whitney test was performed.
(TIF)

Table S1 **Microarray validation by RT-qPCR.** Validation was performed on six transcripts found significantly up- and down-regulated by microarray in the two comparisons NS 2 Unexp vs P and NS2 Exp vs P. 95% confidence intervals were calculated for qPCR fold changes. Significant fold changes between NS2 and P are marked with ¥ and significant fold changes between NS2 and NO are marked with ¤.
(XLSX)

Table S2 **List of significant transcripts commonly found in “NS 2 Exp vs P” and NS 2 Unexp vs P” comparisons.**
(XLSX)

Table S3 **List of** ***CYPs*** **commonly found in both RS and CS comparisons.**
(XLSX)

Table S4 **List of transcripts significantly commonly found in both RS and CS comparisons with fold changes higher than 1.25 times in RS compared to RC fold changes.**
(XLSX)

Table S5 **List of primers used for PCR and qPCR.**
(XLSX)
