# Supplementary figures and images for: Identification of Carboxylesterase Genes Implicated in Temephos Resistance in the Dengue Vector Aedes aegypti
Source: PLoS Negl Trop Dis. 2014 Mar 20;8(3):e2743. doi: 10.1371/journal.pntd.0002743 (PMC3961196; doi:10.1371/journal.pntd.0002743)

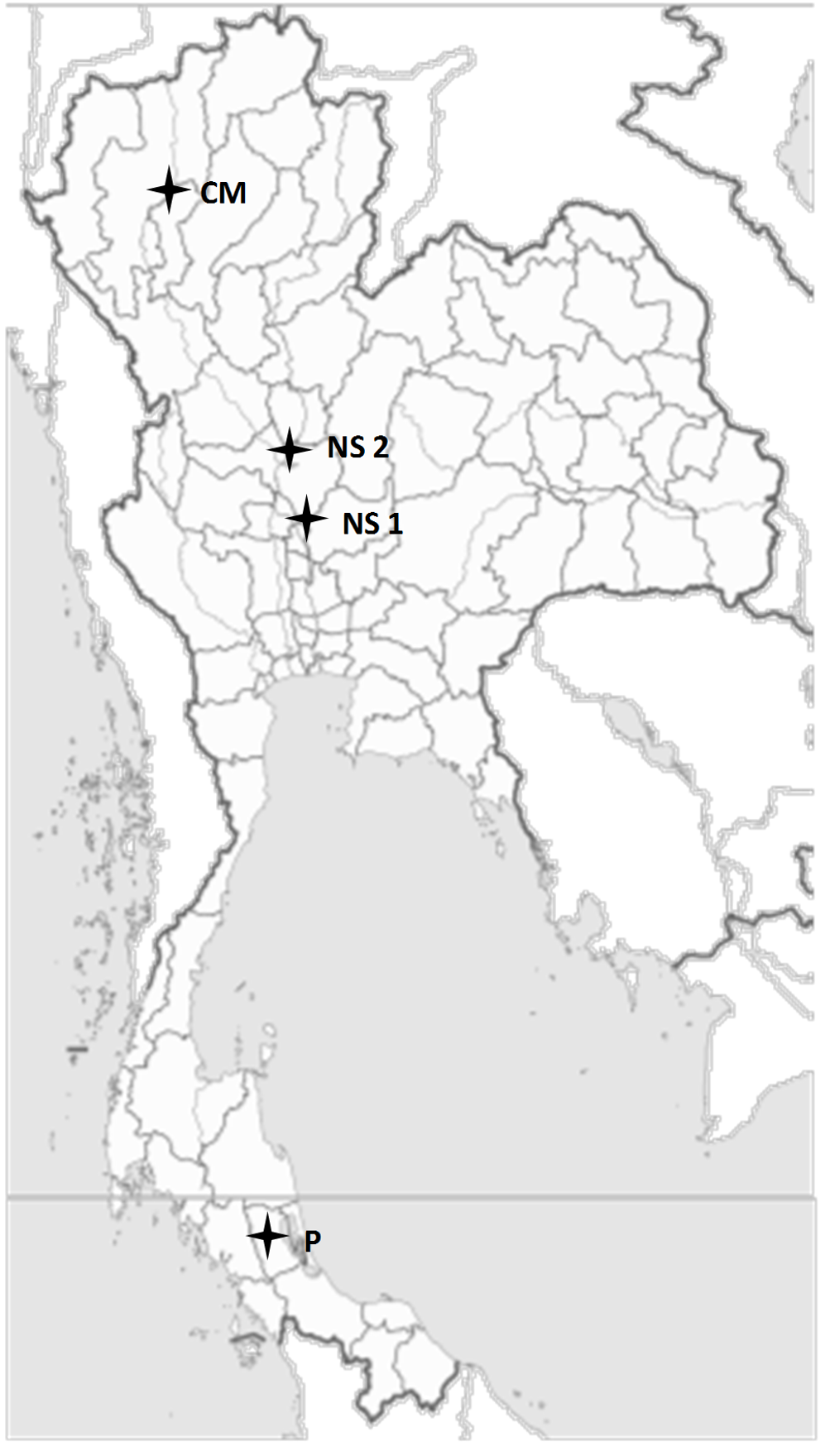

Supplement: Figure S1 — Sampling sites of Aedes aegypti mosquitoes in Thailand. Eggs were collected from four different sites were used: Chiang Mai (CM) (Oct 2011), Nakhon Sawan 1 (NS 1) (March 2012), Nakhon Sawan 2 (NS 2) (March 2012), Phatthalung (P) (September 2012). (TIF) [file pntd.0002743.s001.tif]

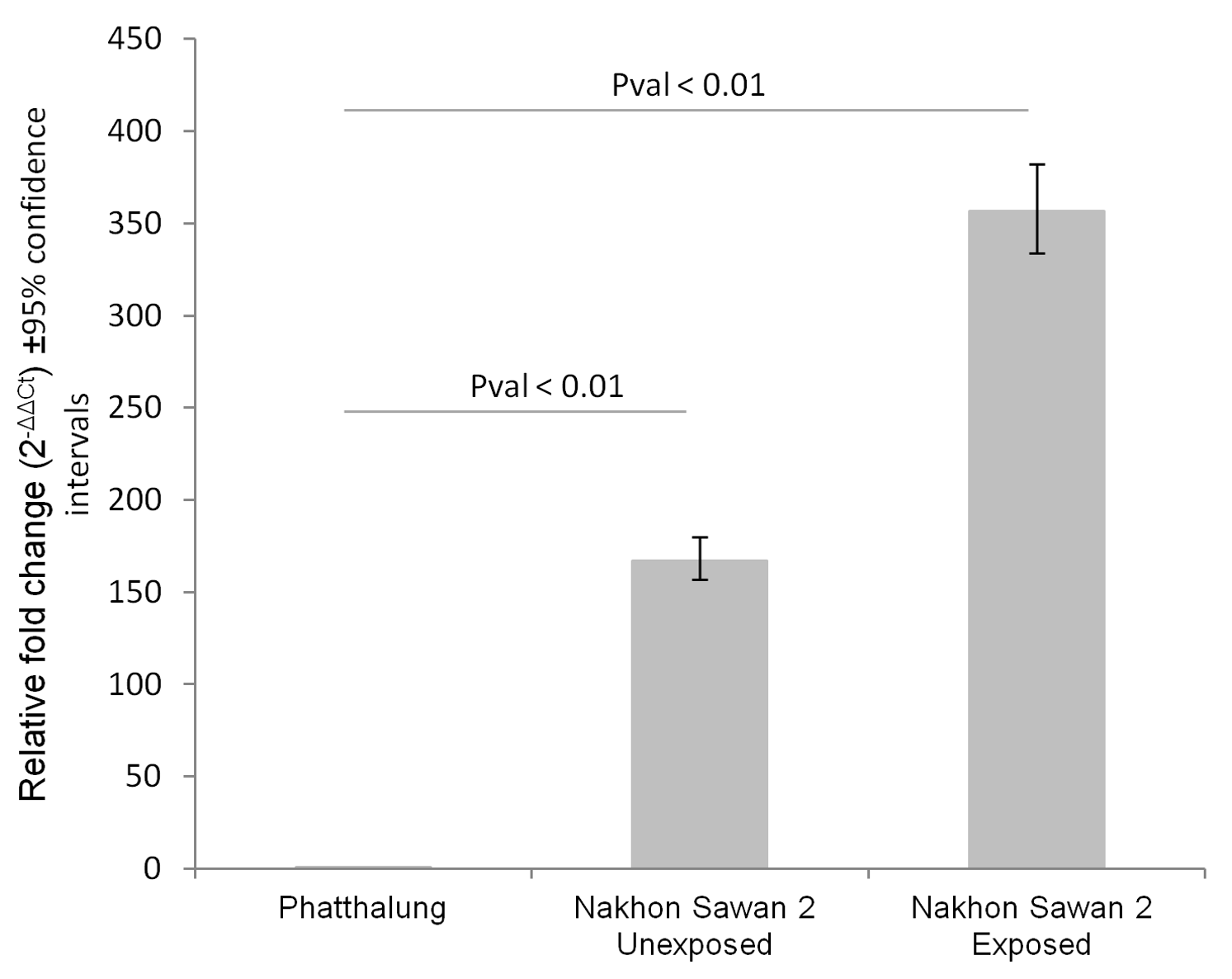

Supplement: Figure S2 — CCEae3a gene copy number analysis. qPCR was conducted on three batches of 8 individual 4th instar larvae gDNA from NS 2 unexposed, NS2 Exposed (larvae survivors of a temephos bioassay inducing more than 80% mortality after 24 hours) and Phatthalung (P). 95% confidence intervals were calculated for qPCR fold changes and a Mann-Whitney test was performed. (TIF) [file pntd.0002743.s002.tif]
